# Immune cell infiltrates as prognostic biomarkers in pancreatic ductal adenocarcinoma: a systematic review and meta‐analysis

**DOI:** 10.1002/cjp2.192

**Published:** 2021-01-22

**Authors:** Andrew J McGuigan, Helen G Coleman, R Stephen McCain, Paul J Kelly, David I Johnston, Mark A Taylor, Richard C Turkington

**Affiliations:** ^1^ The Patrick G Johnston Centre for Cancer Research Queen's University Belfast Belfast UK; ^2^ Centre for Public Health Queen's University Belfast Belfast UK; ^3^ Department of Hepatobiliary Surgery Mater Hospital, Belfast Health and Social Care Trust Belfast UK; ^4^ Department of Tissue Pathology Royal Victoria Hospital, Belfast Health and Social Care Trust Belfast UK; ^5^ Northern Ireland Cancer Centre Belfast Health and Social Care Trust Belfast UK

**Keywords:** pancreatic cancer, prognostic biomarker, immune infiltration, immunohistochemistry, systematic review, meta‐analysis

## Abstract

Immune cell infiltration has been identified as a prognostic biomarker in several cancers. However, no immune based biomarker has yet been validated for use in pancreatic ductal adenocarcinoma (PDAC). We undertook a systematic review and meta‐analysis of immune cell infiltration, measured by immunohistochemistry (IHC), as a prognostic biomarker in PDAC. All other IHC prognostic biomarkers in PDAC were also summarised. MEDLINE, EMBASE and Web of Science were searched between 1998 and 2018. Studies investigating IHC biomarkers and prognosis in PDAC were included. REMARK score and Newcastle–Ottawa scale were used for qualitative analysis. Random‐effects meta‐analyses were used to pool results, where possible. Twenty‐six articles studied immune cell infiltration IHC biomarkers and PDAC prognosis. Meta‐analysis found high infiltration with CD4 (hazard ratio [HR] = 0.65, 95% confidence interval [CI] = 0.51–0.83.) and CD8 (HR = 0.68, 95% CI = 0.55–0.84.) T‐lymphocytes associated with better disease‐free survival. Reduced overall survival was associated with high CD163 (HR = 1.62, 95% CI = 1.03–2.56). Infiltration of CD3, CD20, FoxP3 and CD68 cells, and PD‐L1 expression was not prognostic. In total, 708 prognostic biomarkers were identified in 1101 studies. In summary, high CD4 and CD8 infiltration are associated with better disease‐free survival in PDAC. Increased CD163 is adversely prognostic. Despite the publication of 708 IHC prognostic biomarkers in PDAC, none has been validated for clinical use. Further research should focus on reproducibility of prognostic biomarkers in PDAC in order to achieve this.

## Background

Pancreatic cancer remains a challenging disease, with only small improvements in overall survival rates observed in recent years [1]. Currently, less than 10% of affected patients will survive for 5 years after diagnosis [2, 3]. Pancreatic ductal adenocarcinoma (PDAC) is the predominant subtype of the disease and it is set to become the second most common cause of cancer‐related death in the United States by 2030 [4]. A better understanding of PDAC and more targeted and immune‐therapeutic options are therefore urgently needed.

Traditional TNM staging methods do not reliably predict outcome in PDAC, with many patients experiencing early disease recurrence [5]. As well as indicating survival outcomes regardless of therapy, prognostic biomarkers can also give a valuable insight into the underlying biology and natural history of tumours; for example poorer survival outcomes are observed in patients with *BRAF* mutant colon cancer [6, 7]. Biomarkers identifying individuals at risk of early recurrence following a PDAC diagnosis would be especially useful as they could avoid ultimately futile surgery or identify those in whom neoadjuvant or adjuvant treatment would potentially improve outcomes [5].

Biomarkers assessed by immunohistochemistry (IHC) are particularly helpful, since this technique is widely used in routine clinical practice and its validity in formalin fixed paraffin embedded tissue means that no additional preservation procedures are required [8]. Tissue morphology is also maintained, enabling the location and quality of receptor or gene expression to be determined [8]. The importance of IHC biomarkers evaluating the tumour microenvironment is also increasingly recognised [9]. The use of immune cell infiltration as a prognostic marker superior to conventional TNM staging has been described in colorectal cancer, and many studies have sought to identify similar prognostic scores in PDAC based on immune cell infiltration [10, 11, 12, 13, 14, 15, 16, 17, 18, 19, 20, 21, 22, 23, 24, 25, 26, 27, 28, 29, 30, 31, 32, 33, 34, 35, 36]. There is therefore a pressing need to conduct a systematic review to collate and assess the quality of evidence for IHC‐derived prognostic biomarkers, particularly those assessing the immune cell infiltrate, for PDAC. Previous reviews of this topic require updating and had limitations such as not conducting meta‐analyses [37, 38] or only using a single database to identify articles [5].

The aim of this systematic review is to summarise the quality of published studies investigating the prognostic role of immune based prognostic biomarkers in PDAC and pool the results using meta‐analyses, where possible. The secondary aim is to summarise all other IHC derived prognostic biomarkers in PDAC in order to highlight areas for validation and possible gaps in evidence.

## Methods

This review was reported in accordance with the PRISMA and MOOSE guidelines [39, 40].

### Search strategy

The electronic databases Ovid MEDLINE (US National Library of Medicine, Bethesda, MD, USA), EMBASE (Reed Elsevier PLC, Amsterdam, Netherlands), and Web of Science (Thomson Reuters, New York, NY, USA) were searched on the week beginning 15th October 2018. Medical Subject Heading (MeSH) and keyword searches limited to English language articles were carried out to identify studies investigating IHC biomarkers derived from PDAC tumour tissue and their association with survival. The CONKO‐001 trial, investigating the benefit of adjuvant gemcitabine in PDAC, began recruitment in 1998 [41]. We therefore limited our search strategy to articles published between 1998 and 2018 in an attempt to include all relevant studies performed in the era when adjuvant chemotherapy may have been considered in PDAC patients.

The search strategy identified articles containing at least one MeSH term or keyword related to pancreatic cancer in combination with MeSH terms or synonyms for survival, biomarkers and IHC. The full search terms for each database are available in the supplementary material and the review protocol was registered on the PROSPERO database (CRD 42018115924) [42].

### Study selection and data extraction

Titles and abstracts were reviewed by two authors independently (AJM reviewed all articles; RCT, RSM, DIJ and HGC each reviewed a subset) and any discrepancies were resolved by discussion with a third reviewer. The ‘PICOS’ criteria was used to determine eligibility of included articles:

#### Participants

Individuals of any age with a diagnosis of PDAC. If studies used the term pancreatic cancer it was assumed that this referred to PDAC unless otherwise stated.

#### Intervention

The assessment of biomarkers identified by IHC analysis.

#### Comparators

Comparison was made between different levels of the immunohistochemical biomarkers assessed (e.g., high versus low expression).

#### Outcome

Disease‐free survival (DFS), overall survival (OS), disease‐specific survival (DSS). Disease free, overall and disease‐specific survival were defined as the time between surgery and evidence of recurrence of malignancy, death from any cause and death from PDAC, respectively.

Observational and interventional studies were included if they measured the association between a biomarker identified by IHC and prognosis in patients with PDAC. The initial scope of the systematic review was to include all IHC derived prognostic biomarkers in PDAC. However, a large number of studies were identified so a *post hoc* change was made to the review protocol, whereby only articles which investigated the levels of immune cell infiltration in pancreatic tumour tissue were retained for detailed data extraction, and meta‐analysis where possible. All other prognostic biomarkers from the remaining articles were summarised to provide a broad overview of the available literature.

AJM reviewed the abstracts for all eligible studies to record the prognostic biomarker investigated. Abstracts describing immune response or specific immune cell markers (CD4, CD8, FoxP3, *etc*), and their association with prognosis, were retained for full text review. Data extraction (study population characteristics, marker(s) studied, hazard ratios [HRs], *etc*.) from the articles relating to immune infiltration was performed by AJM and this was checked by a second reviewer (RSM, RCT or HGC) to ensure accuracy. The Newcastle–Ottawa scale (NOS) [43] was used to assess the risk of bias and the quality of study design and data collection was measured using the REMARK guidelines [44].

### Statistical analysis

The association between OS, DSS or DFS in PDAC patients and the level of tumour infiltration by immune cells, identified by IHC, was determined. HRs and corresponding 95% confidence intervals (CIs) were extracted from each publication. If these results were not explicitly stated, Parmar's method [45] was used to derive HR and 95% CI from Kaplan–Meier curves, or 95% CI were calculated from the *P* value using the method described by Altman [46] in any study which only reported this. Results from the multivariable models adjusted for the largest number of confounding factors were pooled, whenever possible.

Random‐effects meta‐analysis was undertaken using RevMan 5.3 software (The Nordic Cochrane Centre, The Cochrane Collaboration, Copenhagen) [47] to calculate pooled HR estimates and the heterogeneity between studies determined by the *I* ^*2*^ statistic. *I* ^*2*^ estimates the proportion of the variance in studies included in the meta‐analysis that is due to heterogeneity between them [48]. Results of this measure of heterogeneity are traditionally described as low (25%), moderate (50%) or high (75%) [49].

For comparability, we evaluated ‘high’ versus ‘low’ immune cell infiltration/expression across studies. The HR and 95% CI from any study reporting associations for ‘low’ versus ‘high’ expression were, therefore, inverted prior to inclusion in meta‐analyses or summary tables. Results reported separately for survival associated with immune cell infiltration of specific tumour areas (e.g. stroma and tumour core) were combined by meta‐analysis prior to inclusion in the main meta‐analysis for specific variables. Publication bias was determined by assessing the symmetry of a funnel plot. Subgroup analysis based on the site of tumour was planned but a lack of relevant studies precluded this.

### Ethical approval

This systematic review summarises previously published data and does not include new human data or tissue that require ethical approval and consent. The authors assume that the studies reviewed were conducted after ethical approval and consent, and in accordance with the Declaration of Helsinki.

### Consent for publication

This manuscript does not contain any individual person's data. All data reported are found in the literature as cited in the text.

## Results

**Figure 1:**
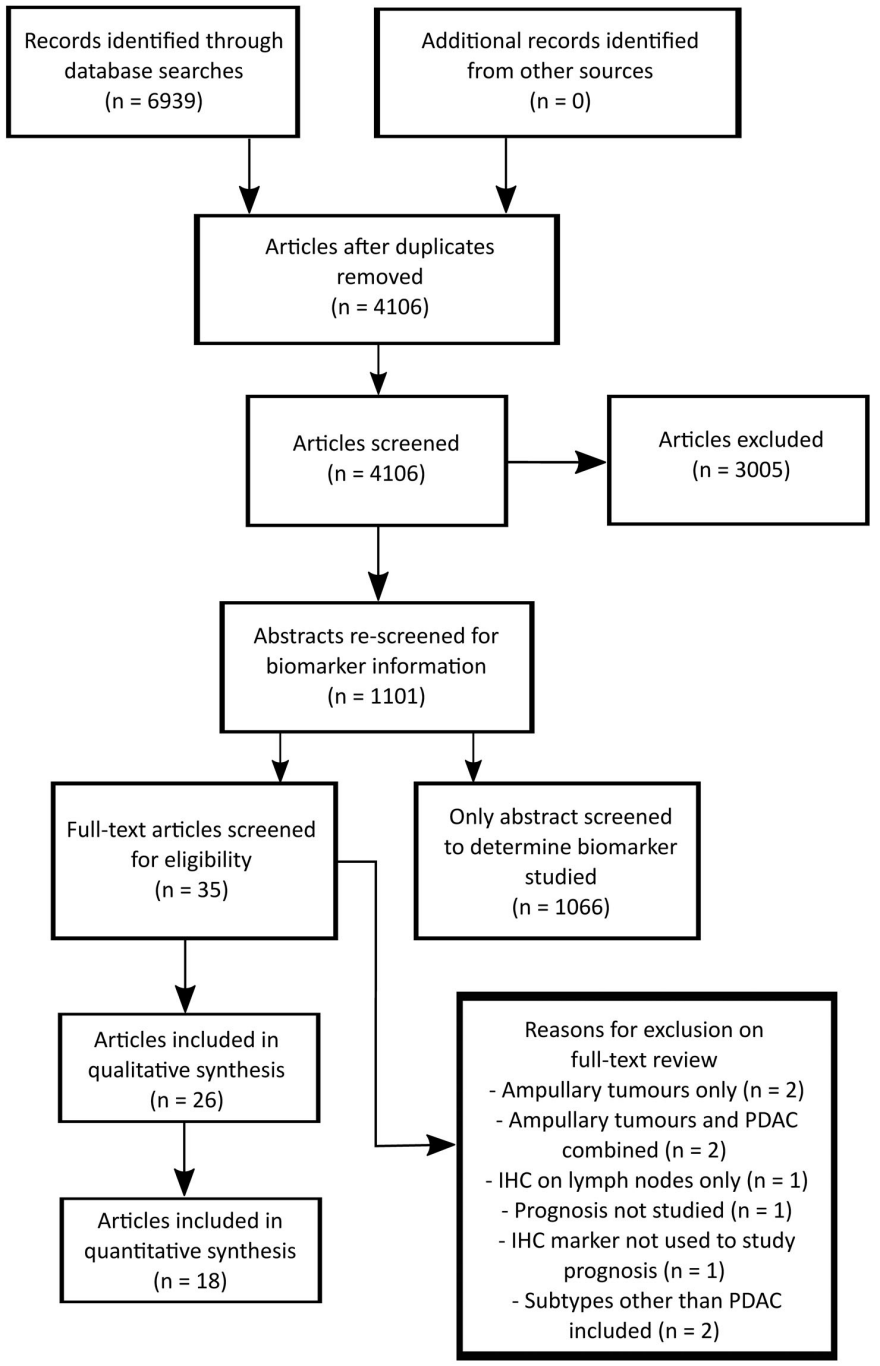
PRISMA flow diagram of study selection.

The PRISMA flowchart [39] of study selection is shown in Figure 1. Searches of the databases identified 6939 articles and 4106 remained after duplicates were removed. Following title and abstract screening, consensus between authors identified 1101 potentially relevant articles eligible for full text review, describing 708 unique prognostic IHC biomarkers in PDAC (see supplementary material, Table S1). p53 was the most frequently investigated biomarker, appearing in a total of 58 papers. However, a total of 483 markers have only been investigated in a single article. The remainder of this review focusses on studies reporting immune cell infiltrate IHC biomarkers and PDAC prognosis.

### Immune cell infiltrate biomarkers

Twenty‐six studies [10, 11, 12, 13, 14, 15, 16, 17, 18, 19, 20, 21, 22, 23, 24, 25, 26, 27, 28, 29, 30, 31, 32, 34, 35, 36] which assessed the association between immune cell markers (CD3, CD4, CD8, CD15, CD20, CD68, CD163, CD204, CD206, FoxP3, programmed cell death 1 [PD‐1], programmed cell death ligand 1 [PD‐L1] and folate receptor β macrophages), identified by IHC in pancreatic tumour tissue, and survival were eligible for qualitative review. The study population characteristics, methodology and factors adjusted for in multivariable modelling are summarised in supplementary material, Table S2.

The majority of studies constructed a TMA using tissue cores from the PDAC tumour core or invasive front; however, the two studies by Diana *et al* [21, 22] used whole mount pancreatectomy specimens for IHC analysis. Hwang *et al* [25] only included tumours arising in the left side of the pancreas (left of the pancreatic neck) and Yoshikawa and colleagues exclusively studied tumours in the head of pancreas [35]. All other authors included tumour tissue from throughout the pancreas and recorded the anatomical distribution. The majority of authors studied tissue from patients treated at their respective institutions. However, Mahajan and colleagues [10] used patients and tissue generated by the ESPAC trials [50, 51, 52] and the CONKO‐001 study [41] was the source of tumour tissue for Lohneis *et al* [15]. Zhang *et al* [19] used a commercially available TMA for their analysis.

Inclusion of patients based on disease stage was diverse. Three studies included stage I–II [11, 28, 29], three stage I–III [12, 26, 27], eight included patients with stage I–IV disease [10, 13, 18, 19, 31, 32, 35, 36] and 12 did not provide information on staging [14, 15, 16, 17, 20, 21, 22, 23, 24, 25, 30, 34]. With regards to resection margin, Liu *et al* [26] and Sugimoto and colleagues [31] only included tumour resections with a microscopically clear margin (R0, >1 mm tumour clearance from nearest margin).

All participants in the Nejati *et al* [16] study and a proportion in the studies by Castino *et al* [20] and Sugimoto *et al* [31] were treated with neoadjuvant chemotherapy prior to resection. The use of adjuvant chemotherapy varied widely between studies, with 10 articles [12, 14, 16, 23, 24, 25, 27, 29, 34, 36] not including any information about the use of adjuvant treatment. A further five studies [13, 18, 19, 20, 30] did not outline the chemotherapy regimen used. In addition, no study excluded death within 30 days following surgery so the OS may have been influenced by peri‐operative deaths which are not truly related to the biomarkers of interest.

The REMARK criteria [44] were used to determine study quality, with one point awarded for each domain up to a maximum of 20. These results are summarised in supplementary material, Table S3. The scores ranged from 12 to 20 with a median of 16.5. Mahajan *et al* [10] was the only study to achieve the maximum score. The lowest scoring studies were penalised for the lack of clearly presented HR and 95% CI and little or no detail regarding the factors considered during multivariable model building. Only Mahajan *et al* [10] and Balachandran and colleagues [13] addressed the issue of sample size, citing the rarity of PDAC and lack of tissue samples as the reasons for the number of patients included.

The NOS was used to determine risk of bias and the results are summarised in supplementary material, Table S4. Only three studies [12, 14, 34] failed to achieve the maximum score of nine, suggesting that there is a low risk of bias within the included articles. A funnel plot (see supplementary material, Figure S1) of all results from the included studies was symmetrical around the line of no effect, implying little influence from publication bias.

### T‐cells

#### CD3 T‐cells

**Figure 2:**
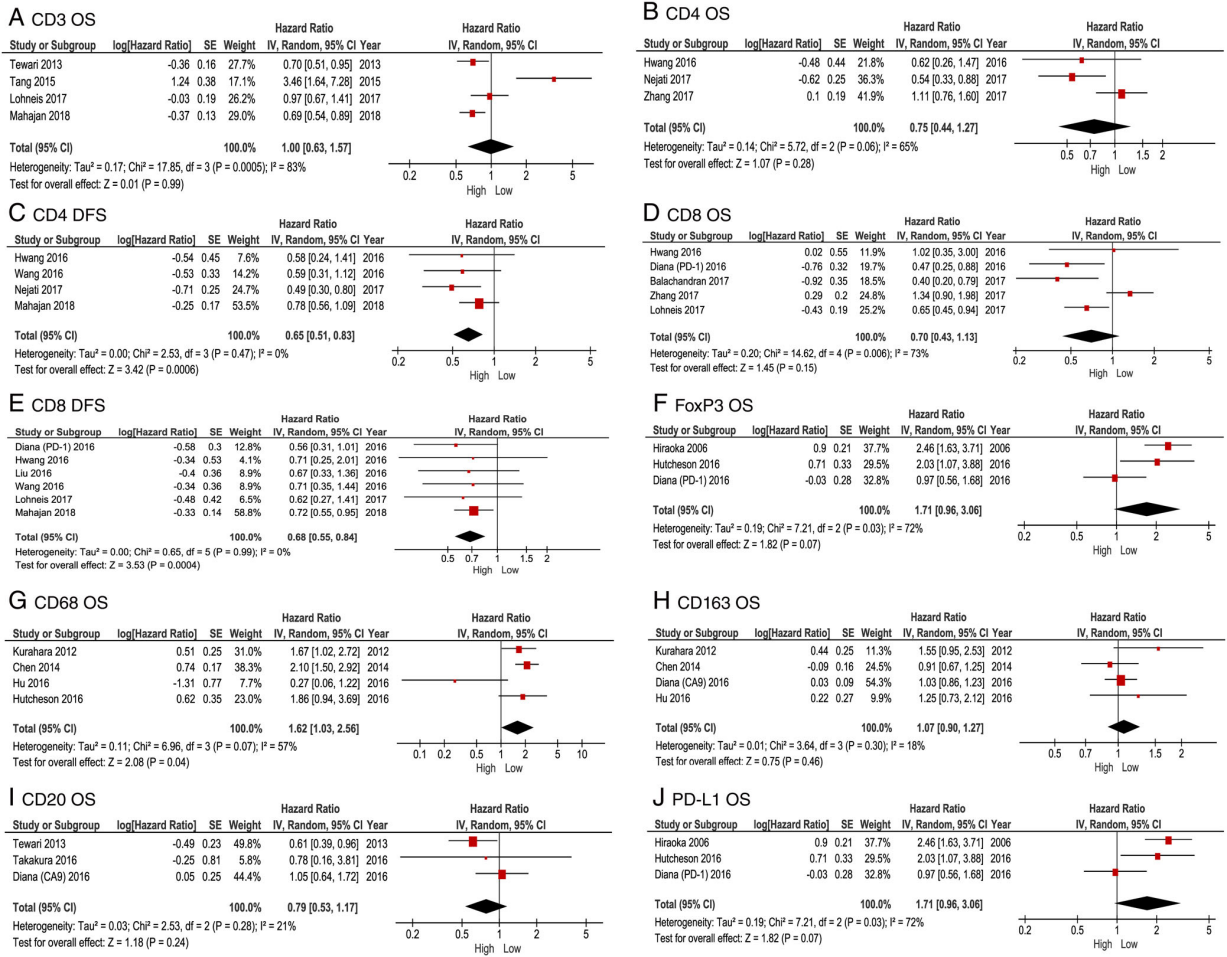
Forest plots of pooled analysis comparing high versus low expression of immune cell infiltration and survival in patients with PDAC.

Four studies [10, 15, 29, 34] investigated the association between CD3 expression and OS in PDAC patients, and all were included in the meta‐analysis (Figure 2A). Pooled analysis revealed no difference in OS for PDAC patients with high, compared to low, levels of CD3 T‐cell infiltration (HR 1.00, 95% CI 0.63–1.57; *p* = 0.99) in their PDAC tumour specimens. Observed heterogeneity was high (*I* ^2^ = 83%, *p* ≤ 0.001).

#### CD4 T‐cells

Six studies [10, 12, 16, 19, 25, 32] investigated the association between CD4 infiltration and prognosis in PDAC, with three [16, 19, 25] included in the meta‐analysis of high versus low CD4 expression and OS (Figure 2B). High levels of CD4 expression were not associated with OS when compared to low CD4 infiltration in PDAC (HR 0.75 95% CI 0.44–1.27; *p* = 0.28). There was moderate heterogeneity between studies (*I* ^2^ = 65%, *p* = 0.06). Four studies [10, 16, 25, 28] were pooled in the meta‐analysis of CD4 expression and DFS (Figure 2C). High versus low CD4 infiltration was associated with improved DFS in PDAC (HR 0.65, 95% CI 0.51–0.83; *p* ≤ 0.001), with no heterogeneity between the four studies (*I* ^2^ = 0%, *p* = 0.47).

#### CD8 T‐cells

The association between CD8 infiltration and survival was assessed in 13 individual papers [10, 11, 12, 13, 15, 16, 19, 22, 25, 26, 28, 32, 34]. Pooled analysis of five studies [13, 15, 19, 22, 25] was not associated with OS in high compared to low CD8 infiltration (HR 0.70, 95% CI 0.43–1.13; *p* = 0.15) in PDAC (Figure 2D). Observed heterogeneity was moderate (*I* ^2^ = 73%, *p* = 0.006). Six studies [10, 15, 22, 25, 26, 28] investigated the association between high compared to low levels of CD8 and DFS. In pooled analysis, high levels of CD8 were associated with longer DFS (HR 0.68, 95% CI 0.55–0.84; *p* ≤ 0.001) in PDAC in comparison with low infiltration, with little evidence of heterogeneity (*I* ^2^ = 0%, *p* = 0.99) (Figure 2E).

Castino and colleagues [20] and Liu *et al* [26] measured DSS as their survival outcome. Consequently, these results could not be pooled with those studies investigating DFS or OS. Castino *et al* [20] found no association between the highest and lowest quartiles of CD8 infiltration and DSS in PDAC (HR 0.69, 95% CI 0.27–1.77; *p* = 0.45). Liu et al [26] found longer DSS with high CD8 infiltration of the stroma when compared to low on multivariable analysis (HR 0.10, 95% CI 0.03–0.31; *p* ≤ 0.001). There was no association between high versus low intra‐epithelial CD8 infiltration and DSS in the Liu study [26] in univariate analysis (HR 0.88, 95% CI 0.49–1.59; *p* = 0.67).

Tewari *et al* [34] and Lohneis and colleagues [15] investigated the association between CD8 infiltration in the tumour stroma only and OS. Consequently, these results could not be reliably pooled with other studies that analysed infiltration more widely in the tumours. Tewari *et al* [34] found no difference in OS with high versus low CD8 infiltration in the tumour stroma (HR 0.68, 95% CI 0.40–1.16; *p* = 0.15). Lohneis and colleagues [15] reported improved OS (HR 0.61, 95% CI 0.23–0.99; *p* = 0.01) and DFS (HR 0.55, 95% CI 0.17–0.93; *p* = 0.002) with high compared to low CD8 infiltration in the stromal area of PDAC tumours. The magnitude of effect estimates reported are broadly similar to that found in the meta‐analysis for DFS.

#### FoxP3 (regulatory T‐cells)

Four studies [18, 22, 24, 26] investigated the association between FoxP3 infiltration and prognosis, three of which [18, 22, 24] were included in a pooled analysis of OS (Figure 2F). There was poorer OS observed in patients with high versus low tumour infiltration of FoxP3, but this did not reach statistical significance (HR 1.71, 95% CI 0.96–3.06; *p* = 0.07). There was moderate heterogeneity between studies (*I* ^2^ = 72%, *p* = 0.03). Only two studies [22, 26] investigated the association between FoxP3 infiltration and DFS, so pooled analysis was not undertaken for this outcome. Diana *et al* [22] found no association between high compared to low FoxP3 and DFS in PDAC (HR 0.90, 95% CI 0.53–1.52; *p* = 0.70). The Liu *et al* study [26] did, however, find worse DFS associated with high versus low FoxP3 infiltration in the intra‐epithelial areas of PDAC (HR 3.39, 95% CI 1.33–8.61; *p* = 0.01) but no association between high FoxP3 in the stroma and DFS (HR 0.58, 95% CI 0.30–1.11; *p* = 0.10).

Liu and colleagues [26] also investigated the association between the location of FoxP3 and DSS. They found no association between high FoxP3 infiltration in the intra‐epithelial (HR 0.96, 95% CI 0.38–2.44; *p* = 0.93) or stromal (HR 0.63, 95% CI 0.29–1.38; *p* = 0.25) PDAC tumour compartments and DSS when compared to low FoxP3.

### Macrophages

#### CD68 (pan‐macrophage marker)

Six studies [10, 21, 23, 30, 35, 36] investigated the association between CD68 infiltration and survival and four [21, 23, 30, 36] were included in meta‐analysis (Figure 2G). There was no association between high compared to low CD68 macrophage infiltration in PDAC and OS (HR 1.07, 95% CI 0.90–1.27; *p* = 0.46) with low heterogeneity between studies (*I* ^2^ = 18%, *p* = 0.30).

In univariate analysis, Hu and colleagues [23] found no association between OS and high or low CD68 infiltration when measured in the tumour stroma and islets separately (stroma HR 1.44, 95% CI 0.85–2.42; *p* = 0.17: islet HR 1.38, 95% CI 0.8–2.38; *p* = 0.25). Diana *et al* [21] and Mahajan *et al* [10] found no significant difference in DFS between high and low CD68 infiltration (HR 1.01, 95% CI 0.85–1.20; *p* = 0.91 and HR 0.83, 95% CI 0.64–1.08; *p* = 0.16 respectively).

Yoshikawa *et al* [35] stained for CD68 infiltration in the tumour core and periphery. They only reported mean survival time and *P* value from univariate analysis but found no difference in DSS or DFS between high and low CD68 levels at either the tumour core or periphery.

#### CD163 (M2 polarised macrophages)

Five studies [21, 24, 27, 30, 36] investigating the association between CD163 and survival were identified and four [21, 24, 30, 36] included in the meta‐analysis of high versus low CD163 infiltration and OS (Figure 2H). This identified worse OS in PDAC with high compared to low CD163 levels in PDAC (HR 1.62, 95% CI 1.03–2.56; *p* = 0.04). Heterogeneity between studies was moderate (*I* ^2^ = 57%, *p* = 0.07).

Hu compared high and low CD163 expression in the tumour stroma or islets with OS [23]. Higher CD163 infiltration in the stroma was associated with worse prognosis (HR 6.35, 95% CI 1.4–28.87; *p* = 0.02) but there was no difference in the levels identified in tumour islets on univariate analysis (HR 1.58, 95% CI 0.88–2.86; *p* = 0.13) [23].

#### CD204 (M2 polarised macrophages)

Two studies [31, 35] investigated the association between CD204 and survival. Sugimoto *et al* [31] found poorer OS (HR 2.17, 95% CI 1.52–3.09; *p* ≤ 0.001) and DFS (HR 1.83, 95% CI 1.30–2.58; *p* = 0.001) with high compared to low levels of CD204 at the periphery of PDAC tumours. Likewise, poorer OS (HR 2.01, 95% CI 1.36–2.96; *p* ≤ 0.001) and DFS (HR 2.05, 95% CI 1.40–2.99; *p* ≤ 0.001) was associated with high CD204 infiltration of the neural plexus in PDAC tumours when compared to low levels. Yoshikawa and colleagues [35] also found shorter DSS (2.78, 95% CI 1.74–4.45; *p* ≤ 0.001) and DFS (HR 1.86, 95% CI 1.16–2.99; *p* = 0.01) in high versus low levels of CD204 infiltration at the periphery of PDAC tumours. There was, however, no difference in DSS when high and low levels of CD204 at the tumour core were compared (HR1.04, 95% CI 0.67–1.59; *p* = 0.87).

#### CD206 (M2 polarised macrophages)

Two studies [10, 28] investigated the relationship between CD206 infiltration and DFS. Mahajan and colleagues [10] found that high infiltration of CD206 was associated with better DFS when compared to low CD206 infiltration in PDAC (HR 0.64, 95% CI 0.47–0.87; *p* = 0.004). However, Wang *et al* [28] found poorer DFS in those with high versus low CD206 levels (HR 4.11, 95% CI 2.09–8.10; *p* ≤ 0.001).

#### Folate receptor β (FRβ) (M2 polarised) macrophages.

Kurahara *et al* [36] was the only study to investigate the association between macrophages expressing FRβ and survival. They found poorer OS in PDAC tumours with high FRβ when compared to low (HR 2.07, 95% CI 1.28–3.36; *p* = 0.003).

### Other immune cells

#### CD20 (B cells)

Five studies [20, 23, 27, 28, 34] investigated the prognostic role of CD20 in PDAC and three [23, 27, 34] were included in the meta‐analysis of CD20 cells in PDAC tumour tissue and OS (Figure 2I). Pooled analysis of these studies shows no association between high CD20 levels and OS (HR 0.79, 95% CI 0.53–1.17; *p* = 0.24). Overall heterogeneity between studies was low (*I* ^2^ = 21%, *p* = 0.28).

Castino *et al* [20] investigated the association between CD20 cells and DSS in PDAC. They found no difference in DSS between the first and fourth quartile of individual tumour infiltrating CD20 cells (HR 1.35, 95% CI 0.40–4.49; *p* = 0.63). When CD20 infiltration was measured in areas of aggregated immune cells (described as ‘tertiary lymphoid tissue’), improved DSS was noted in the highest quartile of CD20 infiltration when compared to the lowest (HR 0.24, 95% CI 0.08–0.71; *p* = 0.01). Wang *et al* [28] found no association between high and low CD20 infiltration and DFS in PDAC (HR 1.54, 95% CI 0.82–2.9; *p* = 0.18).

#### Neutrophils

Two studies [10, 28] investigated the association between neutrophil infiltration and DFS but used different markers. Mahajan *et al* [10] used chloracetate esterase staining and reported no difference in DFS between high and low PDAC tumour infiltration with neutrophils in univariate analysis (HR 0.98, 95% CI 0.78–1.22; *p* = 0.87). Using CD15 as a marker, Wang *et al* [28] found poorer DFS with high levels of CD15 when compared to low in PDAC (HR 2.32, 95% CI 1.21–4.47; *p* = 0.01).

#### CD117 (mast cells)

Wang *et al* [28] determined that high levels of CD117 in PDAC conferred a better DFS when compared to low infiltration (HR 0.34, 95% CI 0.17–0.72; *p* = 0.004).

### Immune checkpoint markers

#### Programmed cell death ligand 1

Four studies [14, 17, 24, 26] investigated the association between PD‐L1 and prognosis. Pooled analysis of three studies [14, 24, 26] (Figure 2J) found expression of PD‐L1 was not associated with OS in PDAC (HR 1.24, 95% CI 0.90–1.72; *p* = 0.19). There was low heterogeneity between studies (*I* ^2^ = 0%, *p* = 0.90).

Tessier‐Cloutier *et al* [17] was the only study to assess the relationship between PD‐L1 and DSS. The authors found significantly worse prognosis in those patients with greater than 10% positively staining PDAC tumour cells compared with tumours with less than 10% of cells positive for PD‐L1 expression (HR 2.05, 95% CI 1.03–3.66; *p* = 0.03).

#### Programmed cell death 1

Diana *et al* [22] reported improved OS (HR 0.46, 95% CI 0.26–0.83; *p* = 0.05) with high expression of programmed cell death 1 (PD‐1) when compared to low in PDAC. There was no association between PD‐1 expression and DFS (HR 0.65, 95% CI 0.40–1.07; *p* = 0.09).

### Immune scores

Tahkola and colleagues [11] sought to calculate an immune score based on whether the tumour core or invasive front contained more than the median number of CD3 and CD8 T‐cells. They found improved OS (HR 0.24, 95% CI 0.12–0.46; *p* ≤ 0.001) and DSS (HR 0.23, 95% CI 0.11–0.45; *p* ≤ 0.001) when the highest and lowest density of immune cell infiltration were compared. Wartenberg *et al* [12] described an ‘immune rich’ subtype with high levels of CD3, CD4 and CD8 T‐lymphocytes, and CD20 B cells, coupled with low infiltration of FoxP3 T‐cells. When compared with an ‘immune escape’ subtype with the inverse immune cell landscape, the ‘immune rich’ group had better OS (HR 0.46, 95% CI 0.29–0.73; *p* ≤ 0.001) and DFS (HR 0.49, 95% CI 0.27–0.78; *p* = 0.004).

Hwang *et al* [25] assessed the prognostic impact of the relative infiltration of regulatory FoxP3 T‐cells and cytotoxic CD8 T‐cells and found reduced OS and DFS (HR 3.58, 95% CI 1.46–8.78; *p* = 0.005: HR 3.06, 95% CI 1.26–7.44; *p* = 0.014 respectively) with a high compared to low FoxP3:CD8 ratio. Fukunaga *et al* [32] reported that tumours with both CD4 and CD8 cell counts greater than the mean had improved OS when compared to those without (HR 0.38, 95% CI 0.16–0.91; *p* = 0.03).

## Discussion

In this systematic review we sought to summarise all of the existing studies of prognostic IHC biomarkers in PDAC, and to perform a meta‐analysis in relation to immune cell infiltration and prognosis. A total of 1101 articles were identified relating to the description of tissue based prognostic biomarkers in PDAC, and these investigated over 700 individual biomarkers. The vast majority were only assessed in a single paper and this is in line with previous criticisms of studies investigating prognostic biomarkers; no systematic approach to the discovery of novel markers and little external validation [53, 54]. Meta‐analysis of included studies investigating the association between immune markers in PDAC found improved DFS with high compared to low CD4 and CD8 infiltration and worse OS with high versus low levels of CD163 macrophages.

Our findings in relation to CD4 and CD8 are in keeping with the prognostic benefit of these immune cell types observed in other tumours [33, 55]. Traditionally, T‐cells have been divided into those with an anti‐tumour phenotype (T‐helper 1 [Th1]) and those whose actions promote cancer progression (T‐helper 2 [Th2]) [56]. CD4 and CD8 are effector T‐cells with recognised Th1 activity and our results suggest that these cells fulfil this role in PDAC, with resultant improved DFS. The theory of ‘cancer immunoediting’ suggests that tumour cells can either be eliminated by the host immune system, remain in a state of equilibrium, with the rate of tumour proliferation matching that of cancer cell destruction, or escape the immune response using various mechanisms of resistance [57]. High levels of CD4 and CD8 cell infiltration may achieve partial elimination or equilibrium in these tumours, resulting in longer DFS. However, any surviving tumour cells may escape the immune response resulting in an immune resistant, more aggressive recurrence. This may explain why a high concentration of these cells was not associated with improved OS.

Pooled analysis of studies investigating the association between CD163 and prognosis found that patients with increased tumour infiltration by CD163 macrophages had shorter OS in PDAC. This is in keeping with studies which found that increased infiltration of CD163 was associated with poorer OS in hepatocellular carcinoma, triple negative breast cancer and follicular lymphoma [58, 59, 60]. Macrophages and their function can be considered on a continuum between classically activated (M1) and alternatively activated (M2) [61]. M2 polarised macrophages, such as those expressing CD163, enable tumour growth and invasion through the promotion of angiogenesis and production of matrix metalloproteinases [61]. With regards to CD68, a pan‐macrophage marker, meta‐analysis did not show any association with prognosis. Overall, our findings suggest that increased tumour infiltration by macrophages with the M2 phenotype was associated with worse survival outcomes and that macrophage polarisation is more significant, in terms of prognosis, than absolute number of these cells recruited to the PDAC tumour. This is also consistent with results from analysis of breast and hepatocellular tumours [58, 60].

Meta‐analysis of the studies investigating FoxP3 and CD20 cell infiltration and prognosis in PDAC did not find any association with OS and this conflicts with findings in other cancers. In ovarian tumours, high FoxP3 T‐cells have been associated with a reduction in Th1 response and worse prognosis [62]. Studies in non‐small cell lung cancer found high infiltration of CD20 in combination with increased CD4 and CD8 cells associated with improved survival [63, 64]. Anti PD‐1/PD‐L1 therapy has proved successful in melanoma [65] and non‐small‐cell lung cancer [66] but results in other tumours have been less promising [67]. Our review also found no association between PD‐L1 expression and OS in PDAC and this would be consistent with the lack of trial evidence in favour of PD‐L1 inhibitors in PDAC. These findings suggest that the immune response to PDAC may differ from other cancers and a greater understanding of this is necessary to enable development of effective, immune‐based therapy in this disease.

This review has several strengths. First, it is the most comprehensive review undertaken to date regarding tissue based prognostic biomarkers in PDAC in terms of breadth of search strategy and number of articles included. The quality and risk of bias in the studies was determined using validated tools, the REMARK guidelines [44] and NOS respectively [43], and pooled analysis of significant results was obtained with low statistical heterogeneity between studies. Funnel plot symmetry and a majority of studies achieving the maximum NOS score suggest a limited effect of publication or other sources of bias. The review was registered at PROSPERO and all *post hoc* changes of protocol were documented to ensure transparency.

Our review was restricted to articles published between 1998 and 2018. The benefit of gemcitabine in advanced PDAC was determined in 1997 [68] and the CONKO‐001 trial [41], comparing adjuvant gemcitabine to observation, began recruitment in 1998. Adjuvant chemotherapy was felt to be an important potential confounder in survival analysis so we wished to include all studies published since adjuvant chemotherapy was instituted.

The main weakness of this review is the variation in the patient populations and tumour analysis methods in the included studies. Results from the two studies by Diana *et al* [21, 22], using whole mount pancreatectomy specimens, may give a wider view of immune cell infiltration throughout the entire tumour. However, their results may not be comparable to those obtained using cores of tumour tissue on a TMA. There was also inconsistency in the tumour regions chosen as the source of cores for the TMA. PDAC is characterised by considerable morphological inter‐ and intra‐tumoural heterogeneity [69]. Studies have demonstrated that this extends to a genomic level, with genetic differences identified even within adjacent tumour glands [70, 71]. It is rational to assume that this variation will also correlate to morphological differences. Differences in prognosis, genetics and immune response (B and T cell infiltration) have been shown in tumours arising in the pancreatic head when compared to those in the body or tail, potentially suggesting different biology depending on the anatomical site of the tumour [72]. This variation in clinical characteristics of included patients limits the validity of these findings to particular subsets of patients, depending on the tumour location, stage of disease and/or completeness of resection. As a consequence, the results cannot be applied to all PDAC patients.

Three studies included patients treated with neoadjuvant chemotherapy prior to resection [16, 20, 31]. Recent studies in breast and lung cancer have shown that neoadjuvant therapy can affect immune cell infiltration in these tumours [73, 74]. This treatment may therefore have had an effect on the immune infiltration in the included studies. Neither Castino *et al* [20] nor Sugimoto and colleagues [31] accounted for neoadjuvant treatment in their multivariate survival analysis and this could also be an important confounder in their results. The differences in adjuvant chemotherapy use between studies must also be taken into account before their results can be applied to a wider PDAC patient population.

The majority of studies included patients and tissue from a single centre only, although two [10, 15] used patient cohorts from the ESPAC and CONKO‐001 trials [41, 50, 51, 52]. Use of results from multi‐centre trial cohorts increases the general applicability of the findings by demonstrating that the marker is robust and not influenced by particular preservation and staining techniques used in different institutions.

The REMARK guidelines recommend the reporting of univariable and key multivariable analysis in terms of HR and 95% CIs [44] and we sought to include multivariable results in our pooled analysis where possible. Five studies [12, 14, 25, 30, 36] only presented univariable analysis and it is therefore impossible to determine if their results represent independent prognostic markers which may out‐perform existing factors which estimate survival. The fact that no study excluded mortality during the peri‐operative period may dilute the effect of the studied biomarkers on OS as this result may have been influenced by factors other than the biomarker of interest. Finally, our search was limited to English language articles; therefore, there is the potential for selection bias due to the inappropriate exclusion of studies only published in other languages.

Whilst no prognostic biomarker has yet been validated for clinical application in PDAC patients, our review has identified over 700 potential markers already investigated and the methodological variation preventing widespread applicability. This review should therefore prove a catalyst for the wider validation of the immune markers already published, to determine their utility as independent prognostic markers in PDAC and enable translation to routine clinical practice. The difficulty of reproducing results in different institutions has been highlighted [75], further reinforcing the need for clear methodology and collaboration between centres in the arena of biomarker discovery and validation. Our findings have also suggested an important role for the immune system in PDAC and further research in this area may yield an important therapeutic breakthrough. Advances in multiplex IHC platforms are enabling the analysis of multiple immune markers simultaneously, and have been investigated in pancreatic, gastric and oesophageal cancer [76, 77, 78, 79]. Multiplex staining and advanced immunoprofiling enable the investigation of biomarker expression and immune cell infiltration, whilst preserving the special organisation of tumour tissue [80]. These techniques have the potential to give a greater insight into the role of immune cell infiltration in cancer than the monostain IHC methods used in the included studies [81].

Whilst this review focussed on the association between immune infiltration and prognosis, there is potential for the markers identified to be used in the selection of checkpoint inhibitors and other immunotherapies. However, to date no immunotherapies or companion diagnostics have been approved for the treatment of PDAC. Although PD‐L1 expression was not associated with prognosis in our pooled analysis, given the success of PD‐L1/PD‐1 inhibitors in other cancers we believe further study and validation of this biomarker should be a priority. The recent FDA approval of the PD‐L1 inhibitor pembrolizumab in advanced cancers with mismatch repair deficiency, regardless of site of origin, highlights the benefit of treatment stratification based on the molecular characteristics of tumours. It is, therefore, imperative that a robust, validated biomarker is developed to ensure PDAC [82] patients who may potentially benefit from these treatments are identified.

Given the widespread use of IHC, the identification of a validated prognostic marker using this technique is an attractive prospect which could be readily applied to current tissue analysis. The heterogeneity of PDAC tumour tissue is a challenge and any sampling protocol would have to be reproducible and enable the collection of representative tissue. ‘Cut points’ to determine ‘high’ versus ‘low’ infiltration would also need to be standardised for the marker to be used in routine clinical practice.

In keeping with other tumour types, high CD4 and CD8 infiltration is associated with improved DFS in PDAC and increased expression of CD163 macrophages is adversely prognostic. Despite the publication of over 700 IHC prognostic biomarkers in PDAC, none has been sufficiently externally validated to enable use in clinical practice. Further high‐quality research is required to focus on reproducibility of prognostic IHC biomarkers, particularly CD4, CD8 and CD163, in PDAC. In order to achieve comparable results and validate these markers, we suggest a standardised, international consensus for the investigation and validation of prognostic biomarkers in PDAC is developed. In line with other cancers, optimal sampling protocols for TMA analysis of PDAC are required to mitigate the effects of morphomolecular heterogeneity [83, 84].

## Author contributions statement

AJM acquired, analysed, and interpreted the data, and drafted the manuscript. HGC designed the review, acquired and interpreted the data, and revised the manuscript. RSM and DIJ acquired the data and revised the manuscript. PJK and MAT analysed the data and revised the manuscript. RCT designed the review, acquired and interpreted the data, and reviewed the manuscript. All of the authors approved the final version for publication and agree to be accountable for all aspects of the work.

## Supporting information


**Search terms**
**Figure S1.** Funnel plot of included studies
**Table S1.** List and frequency of prognostic biomarkers investigated in included studies
**Table S2.** Characteristics of included studies
**Table S3.** REMARK score
**Table S4.** Newcastle–Ottawa score

## Data Availability

All data reported in this manuscript are found in the literature as cited in the text.
