# Supplementary material for: Immune cell infiltrates as prognostic biomarkers in pancreatic ductal adenocarcinoma: a systematic review and meta‐analysis
Source: J Pathol Clin Res. 2021 Jan 22;7(2):99–112. doi: 10.1002/cjp2.192 (PMC7869931; doi:10.1002/cjp2.192)
Supplement: Supplementary file 1 — Search terms Figure S1. Funnel plot of included studies Table S1. List and frequency of prognostic biomarkers investigated in included studies Table S2. Characteristics of included studies Table S3. REMARK score Table S4. Newcastle–Ottawa score [file CJP2-7-99-s001.docx]

**Immune cell infiltrates as prognostic biomarkers in pancreatic ductal adenocarcinoma: a systematic review and meta-analysis**

McGuigan A *et al. J Pathol Clin Res* DOI: 10.1002/cjp2.192

**Supplementary Material**

**Contents:**

**Search terms**

**Figure S1.** Funnel plot of included studies

**Table S1.**  List and frequency of prognostic biomarkers investigated in included studies

**Table S2.** Characteristics of included studies

**Table S3.** REMARK score

**Table S4.** Newcastle-Ottawa score

**Search terms:**

***Medline***

1. pancreatic neoplasms/ or (pancrea* adj3 (neoplas* or malignan* or cancer* or adenocarcinom* or carcinoma* or tumo$r*)).mp.
2. survival/ or exp Survival Analysis/ or Survival Rate/ or Prognosis/ or (surviv* or kaplan-meier or prognos* or predict* or longterm or long-term).mp.
3. exp biological markers/ or pancreatic neoplasms/ge or (biomarker* or marker* or expression* or expressed or overexpress*).mp.
4. immunohistochemistry/ or immunophenotyping/ or (immunohistochem* or immunophenotyp*).mp.
5. 1 and 2 and 3 and 4
6. limit 5 to (english language and yr="1998 -Current")

***Embase***

1. exp pancreas cancer/ or exp pancreas carcinoma/ or (pancrea* adj3 (neoplas* or malignan* or cancer* or adenocarcinom* or carcinoma* or tumo$r*)).mp.
2. exp survival/ or Kaplan Meier method/ or exp prognosis/ or (surviv* or kaplan-meier or prognos* or predict* or longterm or long-term).mp.
3. exp marker/ or gene expression/ or gene overexpression/ or gene expression regulation/ or gene expression profiling/ or genotype/ or gene amplification/ or (biomarker* or marker* or expressi* or expressed or overexpressi* or overexpressed).mp.
4. immunohistochemistry/ or immunophenotyping/ or (immunohistochem* or immunophenotyp*).mp.
5. 1 and 2 and 3 and 4
6. (animal*.hw. or exp animal/) not human/
7. 5 not 6
8. limit 7 to (english language and yr="1998 -Current")
9. limit 8 to (conference abstract or conference paper or conference proceeding or "conference review")
10. 8 not 9

***Web of Science***

1. (TS= ((pancrea* AND cancer) OR (pancrea* AND carcinoma*) OR (pancrea* AND neoplas*) OR (pancrea* AND adenocarcinoma*) OR (pancreas* AND malignan*))) *AND***DOCUMENT TYPES:**(Article)
2. (ts=(prognos* or survival or outcome or 'disease free survival' or 'kaplan meier' or 'kaplan-meier')) *AND***DOCUMENT TYPES:** (Article)
3. (ts=(biomarker* or marker* or 'biological marker*')) *AND***DOCUMENT TYPES:** (Article)
4. (ts= (immunohistochem* OR immunophenotyp*)) *AND***DOCUMENT TYPES:** (Article)


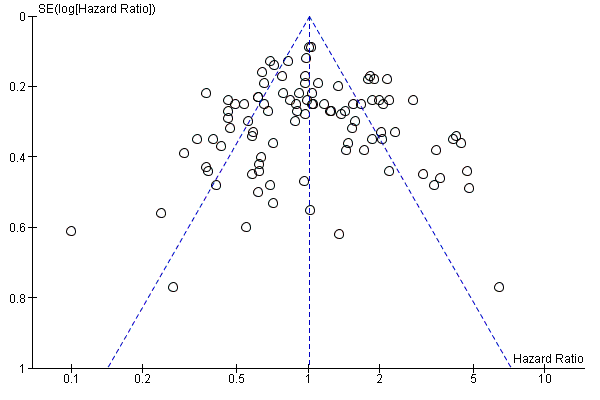


**Figure S1.** Funnel plot of included studies

**Table S1.**  List and frequency of prognostic biomarkers investigated in included studies

| Biomarker | Freq | Biomarker | Freq | Biomarker | Freq | Biomarker | Freq | Biomarker | Freq | Biomarker | Freq | Biomarker | Freq | Biomarker | Freq | Biomarker | Freq |
| --- | --- | --- | --- | --- | --- | --- | --- | --- | --- | --- | --- | --- | --- | --- | --- | --- | --- |
| p53 | 58 | MLH | 8 | FAP | 5 | S100A4 | 4 | KRAS | 3 | Catenin Beta | 2 | Gadd45a | 2 | MMR | 2 | Synaptophysin | 2 |
| MVD/CD31/  CD34 | 37 | MUC1 | 8 | GLI1 | 5 | Stat3 | 4 | Midkine | 3 | Cathepsin L | 2 | GAL3 | 2 | mt-TFA | 2 | SYNDECAN2 | 2 |
| VEGF | 32 | MUC2 | 8 | HOX | 5 | Thymidilate phosphorylase | 4 | MMP7 | 3 | CD105 | 2 | GLP-1R | 2 | NCAM | 2 | TFPI2 | 2 |
| Ki-67 | 28 | MUC5AC | 8 | MUC6 | 5 | (MHC) class I+ | 3 | N-cadherin | 3 | CD147 | 2 | GPC-1 | 2 | NEDD9 | 2 | Toll like r | 2 |
| DPC4/Smad4 | 25 | RRM1 | 8 | Nectin | 5 | Akt | 3 | Nestin | 3 | CD20 | 2 | H3K4me2 | 2 | Neuron specific enolase | 2 | TRAIL-R1 | 2 |
| E-cadherin | 25 | SPARC | 8 | PD-ECGF | 5 | Aldh1 | 3 | Notch1 | 3 | CD40 | 2 | H3K9ac | 2 | NKG2D | 2 | Twist | 2 |
| EGFR | 23 | Survivin | 8 | podoplanin/  D2-40 | 5 | Amphiregulin | 3 | Notch3/4 | 3 | CD44v6 | 2 | H3K9me2 | 2 | nm23-H1 | 2 | VEGFR-1/2 | 2 |
| CD8+ T | 18 | Annexin A1/A2/A8 | 7 | Upar | 5 | BCLx | 3 | PCNA | 3 | CD74 | 2 | HDAC2/3  (3=SIRT1) | 2 | NRP-1 | 2 | YAP1 | 2 |
| HER2 | 18 | Bax | 7 | Wnt | 5 | Beclin1 | 3 | Periostin | 3 | cdh | 2 | HER1 | 2 | OPN | 2 | ZEB1/2 | 2 |
| BCL2 | 16 | Carbonic Anhydrase IX | 7 | B cells | 4 | Chromogrannin A | 3 | PKM2 | 3 | CDX2 | 2 | hsp-27 | 2 | P38 | 2 | 15-PGDH | 1 |
| HIF-1a | 16 | Catenins | 7 | CD68 | 4 | CK20 | 3 | PPAR-gamma | 3 | CEACAM6 | 2 | Hyaluronan | 2 | PAP | 2 | 2F reductase | 1 |
| p16 | 15 | CXCR4 | 7 | cdx2 | 4 | CK7 | 3 | RelA/p65/NF kappa b | 3 | CLAUDIN4 | 2 | ID-1 | 2 | PARP1/2 | 2 | 8-OHdG | 1 |
| hENT-1 | 14 | mdm2 | 7 | CK19 | 4 | c-KIT | 3 | RKIP | 3 | cofilin 1 | 2 | IFNAR-1 | 2 | PDX-1 | 2 | Alpha Intergrin | 1 |
| KLF4 | 14 | MSH | 7 | CD8+ (cytolytic) | 4 | c-Myc | 3 | RXR-a | 3 | Collagen | 2 | IFN-gamma | 2 | Peroxiredoxins | 2 | ABCG2 | 1 |
| MMP-1,2 | 14 | Alpha sm actin | 6 | DLL4 | 4 | CX3CR1 | 3 | SDF1 | 3 | CTGF | 2 | IGF-1R | 2 | PMS2 | 2 | Actinin | 1 |
| COX-2 | 12 | B7-H4 | 6 | DNMT1 | 4 | CXCR7 | 3 | Shh | 3 | CTSB | 2 | IL-10 | 2 | p-mTOR | 2 | Actinin4 | 1 |
| Vimentin | 12 | CD24 | 6 | Fascin | 4 | dendritic cells | 3 | SMAD1/2/3/5/8 | 3 | CTSL | 2 | IL-22/IL-22r | 2 | podocalyxin | 2 | ADAM | 1 |
| CD4+ T | 11 | EpCAM | 6 | FGF/FGFr | 4 | EGF | 3 | Tenascin-C | 3 | CX3CL1 | 2 | IL-6 | 2 | Prox1 | 2 | ADAM8 | 1 |
| CD44v2 | 11 | ERCC1 | 6 | GDNF | 4 | Erk | 3 | Tissue Factor | 3 | CXCl12 | 2 | iNOS | 2 | Ptch1 | 2 | ADAM9 | 1 |
| MMP-9 | 11 | Ezrin | 6 | HDAC1 | 4 | FAS | 3 | AGR2 | 2 | dcr3 | 2 | KGFR | 2 | RACK1 | 2 | Adnab9 | 1 |
| Macrophages | 10 | MUC4 | 6 | HER3 | 4 | GAL1 | 3 | AKT2 | 2 | DJ1 | 2 | L1_CAM | 2 | Rb | 2 | AEG-1 | 1 |
| p21 | 10 | Pan-cytokeratin | 6 | HIF-2a | 4 | H3K18ac | 3 | ARG2 | 2 | Dkk-1/3 | 2 | laminin g 2 | 2 | RCAS1 | 2 | AEP | 1 |
| PDL1/2 | 10 | PTEN | 6 | LAT1 | 4 | HER4 | 3 | ASCT2 | 2 | dpd | 2 | LAPTM4B-35 | 2 | RHAMM | 2 | AHNAK | 1 |
| CD3 | 9 | TGF-beta | 6 | MTA1/2/3 | 4 | HLA-G | 3 | ATG5 | 2 | Endoglin (MVD) | 2 | LC3 | 2 | ROR2 | 2 | ALCAM | 1 |
| CYCLIN D1 | 9 | Thymidylate synthase | 6 | MUC16 (CA125) | 4 | HMGA1 | 3 | ATM | 2 | ER | 2 | LNbeta3 | 2 | RRM2 | 2 | ALDH1A1 | 1 |
| p27Kip1 | 9 | TIMP-2 | 6 | Neutrophils/  CD177 | 4 | IDO | 3 | AXL | 2 | EZH2 | 2 | Maspin | 2 | SIRT | 2 | ALK7 | 1 |
| p-Akt | 9 | B7-H1 | 5 | PD1 | 4 | IL-8 | 3 | B7-1 | 2 | FAK | 2 | MGMT | 2 | Snail | 2 | Alpha-enolase | 1 |
| Tregs/FOXP3 | 8 | B7-H3 | 5 | p-Erk | 4 | IMP3/IGF2BP3 | 3 | BFGF | 2 | FEN1 | 2 | MIB-1 | 2 | SOX9 | 2 | AMBRA1 | 1 |
| CD133 | 8 | Cmet | 5 | PSCs | 4 | KAI1 | 3 | Bif-1 | 2 | FoxM1 | 2 | MICA | 2 | Sp-1 | 2 | ANLN | 1 |
| GLUT-1/3 | 8 | dck | 5 | S100A2 | 4 | KLF9/10 | 3 | BNIP3 | 2 | FoxQ1 | 2 | MKK4 | 2 | SULF1/2 | 2 | AP2-a | 1 |

| Biomarker | Freq | Biomarker | Freq | Biomarker | Freq | Biomarker | Freq | Biomarker | Freq | Biomarker | Freq | Biomarker | Freq | Biomarker | Freq | Biomarker | Freq |
| --- | --- | --- | --- | --- | --- | --- | --- | --- | --- | --- | --- | --- | --- | --- | --- | --- | --- |
| APE1 | 1 | Carbonic anhydrase 2 | 1 | CD5 | 1 | CO-029 | 1 | Enolase 1 | 1 | Gelsolin | 1 | HME | 1 | IL-6R | 1 | LNalpha3 | 1 |
| aPKC | 1 | Carbonic anydrase 1 | 1 | CD51/Integrin-a | 1 | Collagen type 4 | 1 | Ephrin | 1 | Golph3 | 1 | hMENA | 1 | ILK | 1 | LNgamma2 | 1 |
| APN/CD13 | 1 | c16orf74 | 1 | CD55 | 1 | Cortacin | 1 | Ephrin r A4 | 1 | GPC3 | 1 | HMGA2 | 1 | Integrin alpha3/6 | 1 | Lox1 | 1 |
| AR | 1 | CA19-9 | 1 | CD56 | 1 | COUP-TF1 | 1 | EPO/EPOr | 1 | gpr54 | 1 | HMGB1 | 1 | IRF1,2 | 1 | LRP | 1 |
| ARHGEF4 | 1 | Calpain1 | 1 | CD57 | 1 | CRKL | 1 | ERP29 | 1 | gpr87 | 1 | HMGB2 | 1 | ITGB4 | 1 | LTBP2 | 1 |
| Artemin | 1 | Calpastatin | 1 | CD97 | 1 | CRMP4 | 1 | ESRP1 | 1 | Granzyme B | 1 | HNF-1b | 1 | JAGGED1 | 1 | Mac-2-BP | 1 |
| ASPP2 | 1 | Calponin2 | 1 | CDC20 | 1 | CTHRC | 1 | ETS1 | 1 | Gremlin 1 | 1 | HOXA13 | 1 | Jak | 1 | Mad2 | 1 |
| ASS1 | 1 | Calreticulin | 1 | CDC42 | 1 | CTLA4 | 1 | FABP4 | 1 | GRK2 | 1 | H-ras | 1 | JAM-A | 1 | MAP4K | 1 |
| aTubulin | 1 | CAP1 | 1 | CDK4 | 1 | CXCR1 (IL-8r) | 1 | Factor H | 1 | GRK9 | 1 | HSF1 | 1 | KCNN | 1 | MAPK | 1 |
| AuroraA | 1 | Cap43 | 1 | CDK9 | 1 | Cyclin E | 1 | FAS L | 1 | GRO-alpha | 1 | hsp-70 | 1 | KDM4 | 1 | Mast cells | 1 |
| B1 integrin | 1 | Capain2 | 1 | CDKN2A | 1 | CyclinB1 | 1 | FAT10 | 1 | GRP78 | 1 | HSPA2 | 1 | Keap1 | 1 | maz | 1 |
| B2M | 1 | CapG | 1 | Cdse1 | 1 | DCC | 1 | FBXW7 | 1 | GTR1 | 1 | HUGL-1 | 1 | KGF | 1 | mct4 | 1 |
| B7-2 | 1 | Caspase3 | 1 | CEA | 1 | DCTD | 1 | Fdxr | 1 | H2AK119Ub1 | 1 | HVEM | 1 | KIAA1199 | 1 | MDIG | 1 |
| B7-DC | 1 | Catenin Gamma | 1 | CEACAM1 | 1 | DDR1 | 1 | Ferroportin | 1 | H3K27Me3 | 1 | HYAL1 | 1 | Kindlin 2 | 1 | MDR | 1 |
| BAF250 | 1 | Caveolin1 | 1 | CEACAM5 | 1 | DDX3 | 1 | Fhit | 1 | H3K4me3 | 1 | ICAM-1 | 1 | Kindlin2 | 1 | MDR1 | 1 |
| BAG1 | 1 | Cbl-b | 1 | CES2 | 1 | DIXDC1 | 1 | Fibronectin | 1 | H4K12ac | 1 | IEX1 | 1 | KL6 | 1 | MEF2D | 1 |
| Bak | 1 | CBX7 | 1 | cFLIP | 1 | DMNT2 | 1 | Folate R alpha | 1 | HAI1 | 1 | IFIT | 1 | KLF6 | 1 | Mesothelin | 1 |
| BCRP | 1 | CCDC34 | 1 | c-fos | 1 | DNMT3 | 1 | FosB | 1 | HAS2 | 1 | IFNAR-2c | 1 | KLF8 | 1 | Metastin | 1 |
| Beta2-ar | 1 | CCDC88A | 1 | CHD7 | 1 | DSG1,2,3 | 1 | FoxC1 | 1 | Hcnt | 1 | IGFBP3 | 1 | KLK10 | 1 | MICB | 1 |
| BHLHB2 | 1 | CCNG2 | 1 | CHST15 | 1 | DST | 1 | FoxO3a | 1 | HDAC4 | 1 | IGFBP7 | 1 | KLK6 | 1 | miR-10a-5p | 1 |
| BIRC1 | 1 | CCR7 | 1 | CIP2A | 1 | Dysadherin | 1 | FoxP1 | 1 | HDAC6 | 1 | IHH | 1 | KOC | 1 | miR-221 | 1 |
| Bmal | 1 | CD10 | 1 | c-jun | 1 | Dystroglycan | 1 | FRAT1 | 1 | Hdgf | 1 | IkappaB kinase | 1 | KRT19 | 1 | miR-301a-3p | 1 |
| BMI1 | 1 | CD138 | 1 | CK19 | 1 | E2F-1 | 1 | FRS2 | 1 | Heparinase | 1 | IL-1 Beta | 1 | LAMP | 1 | miR-335 | 1 |
| BMP | 1 | CD146 | 1 | CK5/6 | 1 | ECH1 | 1 | FXR | 1 | Hepcidin | 1 | IL-11 | 1 | Latexin | 1 | miR-337 | 1 |
| BMPR1A | 1 | CD151 | 1 | CLAUDIN1 | 1 | ECSOD | 1 | FZD1 | 1 | HES1 | 1 | IL-12 | 1 | LC3B | 1 | MLKL | 1 |
| BRCA1 | 1 | CD154 | 1 | CLAUDIN7 | 1 | Edil3 | 1 | GAB2 | 1 | HEY1 | 1 | IL-13 | 1 | Ldha | 1 | MMP19 | 1 |
| BRCA2 | 1 | CD155 | 1 | CLIC1 | 1 | EF1A2 | 1 | GaINAc | 1 | Hgf | 1 | IL-17 | 1 | LDHB | 1 | MMP20 | 1 |
| BRM | 1 | CD163 | 1 | CLIC4 | 1 | EGFL | 1 | GaINAc-t3 | 1 | HINT2 | 1 | IL-18 | 1 | Lgr5 | 1 | Moesin | 1 |
| BTG1 | 1 | CD206 | 1 | Clusterin | 1 | EGFRrp | 1 | Gal9 | 1 | Hiwi | 1 | Il-1R | 1 | LI cadherin | 1 | Mortalin | 1 |
| BubR1 | 1 | CD36 | 1 | CNKSR1 | 1 | EMMPRIN | 1 | GCSF | 1 | HK2 | 1 | IL-2 | 1 | LMO2 | 1 | mr-1 | 1 |

| Biomarker | Freq | Biomarker | Freq | Biomarker | Freq | Biomarker | Freq | Biomarker | Freq | Biomarker | Freq | Biomarker | Freq |
| --- | --- | --- | --- | --- | --- | --- | --- | --- | --- | --- | --- | --- | --- |
| MRE11 | 1 | Nucleolin | 1 | PLA2 | 1 | RASSF6 | 1 | SIRT 1-7 | 1 | TIM3 | 1 | WT1 | 1 |
| MRP | 1 | NUMB | 1 | PLAC1 | 1 | RBM3 | 1 | Six1 | 1 | TIP30 | 1 | WTAP | 1 |
| MRP1 | 1 | Nurr1 | 1 | plgR | 1 | RECK | 1 | SKI | 1 | TLE1 | 1 | Wwox | 1 |
| MSI2 | 1 | N-WASP | 1 | PLK1 | 1 | Reg | 1 | Skp2 | 1 | TLR4 | 1 | XAF1 | 1 |
| mt2-mmp | 1 | OLMF4 | 1 | PLXNA1 | 1 | Reg3a | 1 | Slug | 1 | TM4SF1 | 1 | XB130 | 1 |
| MTDH | 1 | OX40 | 1 | PMEBA | 1 | Reg4 | 1 | SMAD6 | 1 | TNIK | 1 | XIAP | 1 |
| MTHFD2 | 1 | p120ctn | 1 | PML | 1 | Resistin | 1 | SMAD7 | 1 | Topo2 | 1 | yb-1 | 1 |
| MTI-MMP | 1 | p62/ubiquitin | 1 | PNMAL1 | 1 | RET | 1 | Smica | 1 | TRAF6 | 1 | YKL-40 | 1 |
| MTSS1 | 1 | p63 | 1 | PP4c | 1 | RGS16 | 1 | smo | 1 | TRAIL-R2 | 1 | YY1 | 1 |
| MUC13 | 1 | PACK1 | 1 | PPM1A | 1 | RGS6 | 1 | SOX18 | 1 | Transgelin | 1 | ZNF185 | 1 |
| MUC17 | 1 | PAK4 | 1 | PR | 1 | RhoGDI2 | 1 | SOX4 | 1 | Tricellulin | 1 | klk7 | 1 |
| MUC20 | 1 | palladin | 1 | pRb | 1 | Ring1B | 1 | Src | 1 | TRIM29 | 1 | RhoA | 1 |
| Muscarinic 3 | 1 | PAR-4 | 1 | PRELP | 1 | RON | 1 | Stat1 | 1 | TRIM31 | 1 |  |  |
| MVD (F8mab) | 1 | PDGFR | 1 | Profilin1 | 1 | RunX | 1 | Stat4 | 1 | TROP2 | 1 |  |  |
| MYBL2 | 1 | PDPN | 1 | prp | 1 | RXR-b | 1 | Stathmin | 1 | TSC | 1 |  |  |
| NAC1 | 1 | PEDF | 1 | PRR11 | 1 | RXR-g | 1 | STML2 | 1 | TSLP | 1 |  |  |
| NDRG2 | 1 | Pepsinogen C | 1 | ps2 | 1 | S100A11 | 1 | STMN1 | 1 | TSPAN1 | 1 |  |  |
| Nec2 | 1 | persephin | 1 | PSCA | 1 | S100a6 | 1 | Sulfiredoxin | 1 | UbcH10 | 1 |  |  |
| Neogenin | 1 | PFTK1/CDK14 | 1 | psg1 | 1 | Satb-1 | 1 | Syndecan3 | 1 | UCH/PNI marker | 1 |  |  |
| Neurturin | 1 | PG-1 | 1 | PSMA | 1 | SAV1 | 1 | Synuclein-g | 1 | UGP2 | 1 |  |  |
| NF45 | 1 | PGAM | 1 | PSME3 | 1 | SCF | 1 | Tbet | 1 | ULBP | 1 |  |  |
| NFE2L3 | 1 | p-glycoprotein | 1 | PTN | 1 | SCGB1D2 | 1 | TBK1 | 1 | UNC5H3 | 1 |  |  |
| NFR5A2 | 1 | PGP9.5 | 1 | puma | 1 | Sel1L | 1 | Tbx3 | 1 | USP22 | 1 |  |  |
| NGF | 1 | PHD1 | 1 | PXR | 1 | SEMA3A | 1 | TBX4 | 1 | USP9X | 1 |  |  |
| NIBRIN | 1 | PHD2 | 1 | Rab11 | 1 | SFRP5 | 1 | TFAP2C | 1 | VASH2 | 1 |  |  |
| NNMT | 1 | PHD3 | 1 | RAB27A | 1 | SHMT2 | 1 | TFF1 | 1 | Vav3 | 1 |  |  |
| NQO1 | 1 | PHDGH | 1 | RAB27B | 1 | SHP2 | 1 | Th17 | 1 | VCP | 1 |  |  |
| NRF2 | 1 | PIM1 | 1 | RAB5 | 1 | SIAH | 1 | Thymidine kinase 1 | 1 | VTDB | 1 |  |  |
| NSC | 1 | PINCH | 1 | RAD50 | 1 | Siayl-lewis x | 1 | TI platelets | 1 | WIF1 | 1 |  |  |
| N-Syndecan | 1 | PITX1 | 1 | RASSF1a | 1 | SIMA | 1 | Tiam1 | 1 | WISP1 | 1 |  |  |

**Table S2.** Characteristics of included studies

| Author (year) | Location | Median age | % Male | n PDAC | Stage | Follow-up | Marker stained | Antibody | Measure | Cut-point | Source of core(s) | Location of marker | Neoadjuvant treatment (n) | Adjuvant treatment (n) | Survival outcome | Multivariable model |
| --- | --- | --- | --- | --- | --- | --- | --- | --- | --- | --- | --- | --- | --- | --- | --- | --- |
| Mahajan (2018) | UK, Germany | 64 | 58.4 | 385 | I-IV | 60 months (max) | CD3, CD4, CD8, CD68, CD206, αSM actin, tp 1 collagen, neutrophils | Mouse, rabbit | High/Low | ‘cut-off finder’ NIH software  CD3 high 216 cells/mm^2^  CD4 high 89 cells/mm^2^  CD8 high 160 cells/mm^2^  CD68 high 71 cells/ | TC | S | - | Gemcitabine (195),  5-fu/folinic acid (190) | DFS | Stromal composition, CD3, CD4, CD8, CD68, CD206, nodal status, resection margin, T stage |
| Tahkola (2018) | Finland | 66.9  (Mean) | 52.8 | 108 | Ia-IIb | 60 months (max) | CD3, CD8, MLH1 | - | ‘Immune score’ (IS 0-4 = low – high density of immune cell infiltration) | CD3 TC 527 cells/mm^2^  CD3 IF 674 cells/mm^2^  CD8 TC 297 cells/mm^2^  CD8 IF 320 cells/mm^2^ | TC, IF | IE, S | (0) | Gemcitabine or 5-fu-leucovorin (94) | OS, DFS | Age, stage, IS, grade, gender, peri-neural invasion |
| Wartenberg (2018) | Switzerland | 62.8 | 63.6 | 110 | I-III | 35 months (max) | CD3, CD4, CD8, CD20, PD-L1, p63, RHAMM, MLH1, PMS2, MSH2, MSH6 | - | % positive | PD-L1 – 0=0%-1%, 1= ≥1%, 2= ≥5%, 3=≥10%, 4=≥25%, 5≥50% Immune cells – visual estimation and digital analysis  RHAMM – ROC analysis | TC, IF | IE, S | (0) | - | OS, DFS | - |
| Balachandran (2017) | USA | 76 | 52 | 96 | I-IV | 12 years (max) | CD3, CD8, mDC, Treg (FoxP3), macrophages, B cells, MHC1, granzyme B | Rabbit | Staining intensity | - | TC | - | (0) | Regimen not-specified (66) | OS | Chemotherapy, stage, margin involvement, CD3-CD-granzyme B density |
| Imai (2017) | Japan | 68.2  (Mean) | 63.9 | 36 | - | 60 months (max) | HLA1, HLA-DR, PD-L1, PD-1 | - | High/Low (HLA1, PD-1),  Pos/Neg (HLA DR, PD-L1) | Positive=>5% of cells stained | - | - | - | - | OS, DFS | - |
| Lohneis (2017) | Germany | 62 | 58.1 | 165 | - | - | CD3, CD8, CD103 | - | Number, High/Low, Ratio IE:total | ‘Cut off finder’  CD3 105 cells CD8 42 cells  CD8 S 25 cells CD103 34 cells | TC | IE, S | - | Gemcitabine (83) | OS, DFS | Grade, treatment arm (Chemo vs observation), T stage, N stage, margin involvement, CD8 |
| Nejati (2017) | USA | 63.5 | 61.0 | 136 | - | 32.5 months (median) | CD4, CD8, FoxP3 | Mouse | High/Low | 75^th^ percentile of positive cells  CD4 0.88%, CD8 1.44%, FoxP3 0.82% | TC | - | Regimen non-stated (136) | - | OS, DFS | CD4, CD8:FoxP3 ratio, N stage, tumour regression grade |
| Tessier-Cloutier (2017) | Canada | 66.4 | 55.2 | 252 | - | 16 years | PD-L1 | Rabbit | % positive | Percentage of cells | TC | IE, S | - | ‘Pyrimidine nucleoside analog’ (74) | DSS | Age, gender, adjuvant chemotherapy, grade, LVI, PNI, N stage, resection status |
| Zhang (2017) | China | - | 60.1 | 143 | I-IV | 7 years (max) | CD4, CD8 | Rabbit | High/Low  Yes/No IE | Mean cell count  IE CD4/CD8 >3 = Yes | TC | IE, S | - | ‘Conventional chemotherapy’ | OS | Age, stage, IE CD8 |
| Castino (2016) | Italy | - | - | 104 | - | 13 months (mean) | B cells (CD20), CD8, PD-1GC | Mouse | CD20, CD8 - % immune-reactive area (quartiles),  PD-1GC – Yes/No | Median immune reactive area –  CD20-TLT 3.72%,  CD20-TIL 0.41%,  CD8-TIL 0.61% | TC | TLT, S | Regimen non-specified (14) | Regimen not-specified (69) | DSS | Age, nodal involvement, grade, CD20-TLT, CD20-TIL, CD8-TIL |
| Diana (2016) | UK | 65 | 53.1 | 145 | - | 69 months (max) | CD8, FoxP3, PD-1, PD-L1 | - | High/Low | Median | Whole mount pancreatetomy | IE, S, P | (0) | Gemcitabine +/- capcetabine | OS, DFS, LPFS, DMFS | Age, gender, tumour site, T stage, N stage, grade, operative procedure, PNI, LVI, chemotherapy, CD8, FoxP3, PD-1, PD-L1. |
| Diana (2016) | UK | 65 | 51.4 | 141 | - | 69 months (max) | CD68 (pan macrophage), B cells (CD20) | - | High/Low | Median | Whole mount pancreatectomy | IE, S, P | (0) | Gemcitabine +/- capcetabine | OS, DFS, LPFS, DMFS | Age, gender, tumour site, T stage, N stage, grade, operative procedure, PNI, LVI, chemotherapy, CD68, CD31, CD20 CAIX |
| Hu (2016) | China | - | 63.6 | 88 | - | 90 months (max) | CD68 (pan macrophage) CD163 (M2 macrophages) | - | High/Low | High >40 cells/mm^2^ | TC, ANT | S, I | - | - | OS | N stage, nuclear grade, CD163 stroma, CD163 islet and stroma |
| Hutcheson (2016) | USA | 65 | 54 | 223 | - | 60 months (max) | CD163 (M2 macrophages, FoxP3, PD-L1 | - | High/Low – CD163, FoxP3  Positive – PD-L1 | High = >Median % positive (CD163, FoxP3)  Positive = >10% positive (PD-L1) | TC, IF | IE, S | - | - | OS | Grade, N stage |
| Hwang (2016) | South Korea | 62.4 (mean) | 70 | 30 (left sided tumours only | - | 94 months (max) | CD3, CD4, CD8, FoxP3, granzyme B | - | High/ Low  Ratio FoxP3: other markers | Median count/4 x400 fields  CD3 256  CD4 160  CD8 115  Granzyme B 24  FoxP3 28  Rel. ratio  FoxP3:CD3 0.111  FoxP3:CD4 0.169  FoxP3:CD8 0.026  FoxP3:Granzyme B 0.110 | - | - | (0) | - | OS, DFS | - |
| Liu (2016) | China | 61 | 73.9 | 92 | I-III | 36 months (max) | CD4, FoxP3, CD8 | Mouse | High/ Low | Median  IE CD4 8.20  S CD4 15.34  IE CD8 10.23  S CD8 25.45  IE FoxP3 3.15  S FoxP3 2.02 | - | IE,S | 0 | Gemcitabine + Oxaliplatin | DSS, DFS | Grade, LVI, S CD8, IE FoxP3, S FoxP3 |
| Wang (2016) | China | 61.3 | 59.5 | 284 | I-IIb | 72 months (max) | CD4, CD8, FoxP3, CD20, CD68, CD163, CD206, CD15, CD35 | - | High/low | ‘X-tile’ or  median | TC | - | - | Gemcitabine or 5-flurouracil (201) | DFS | CA 19.9, Ki67 >14%, tumour >3cm, T stage, N stage, microvascular invasion, CD4, CD8, CD117, CD15, CD20, CD206 |
| Takakura (2016) | Japan | 66.5 | 57.1 | 28 | I-III | - | CD20  CD66b, CD163 | - | High/Low/ Negative | Median of the mean cell count in three low power fields | TC | IF | (0) | - | DSS | Serum leucocyte count, serum neutrophil count, serum lymphocyte count, NLR, CD163 |
| Tang (2015) | China | - | 68.2 | 66 | I-II | 40 months | Gal-1, CD3 | Mouse | Weak/Strong staining intensity | 0% cells positive = negative, 1-29% = weak, >30% = strong | - | S | - | - | OS | T stage, N stage, PNI, stage, differentiation, GAL1 intensity, CD3 intensity |
| Chen (2014) | China | 58.5 | 62.9 | 107 | - | 60 months | CD68, CD163 | Mouse | High/Low | Mean absolute count from 3 high power fields (x400)  CD68 TC 30.5  CD68 P 71.5  CD163 TC 21.0  CD163 P 45.0 | TC, P | IE, S | - | Regimen not-specified (24) | OS | - |
| Sugimoto (2014) | Japan | 65 | 62.9 | 170 | I-IV | 48 months (max) | CD204 | Mouse | % area High/low | Median  Plx CD204 0.57%,  P CD204 3.34% | TC, P | P, plx | Regimen not-specified (4) | Gemcitabine (40), S1 (10), Gem+S1 (6), 5-fu+cisplatin (4) | OS, DFS | Adjuvant chemotherapy, CEA level, Tumour >3cm, mod-severe LVI, P CD204% high, Plx CD204% high |
| Tewari (2013) | UK | 65 | 66.7 | 81 | - | 42 months (median) | CD3, CD8, CD20 | - | High/Low | ‘X tile’ software and ROC  IE CD3 28.3 cells/mm^2^  S CD3 132.0 cells/mm^2^  IE CD8 11.8 cells/mm^2^  S CD8 48.4 cells/mm^2^  CD20 84.6 cells/mm^2^ | TC | IE, S | - | - | OS | - |
| Kurahara (2012) | Japan | 67 | 68.4 | 76 | I-IV | 80 months (max) | FR β macrophages, CD68, CD163 | Mouse, rabbit | High/Low | Median cell count | TC, IF | IE, PV | (0) | - | OS | T stage, N stage, neural invasion, FRβ |
| Yoshikawa (2012) | Japan | 64 | 59.8 | 107 (HOP only) | I-IV | 60 months (max) | CD68, CD204 | Mouse | High/Low | Median  TC CD68 3.65%,  P CD68 9.92%,  TC CD204 1.64%,  P CD204 3.38% | TC, P | - | (0) | Intraoperative radiotherapy,  Gemcitabine | DSS, DFS | N stage, tumour >3cm, serosal involvement, extrapancreatic neural invasion, CD 204, P CD204 |
| Hiraoka (2006) | Japan | 63 | 57.6 | 198 | I-IV | 20 months (median) | FoxP3 | Monoclonal anti-human | High/Low | Average proportion of FoxP3 in CD4 lymphocytes | TC, S | - | (0) | ‘Standard therapy appropriate for clinical stage’ (198) | OS | Age, sex, T stage, N stage, M stage, grade, resection margin, FoxP3 |
| Fukunaga (2004) | Japan | 62 (mean) | 56.2 | 80 | I-IV | 98 months (max) | CD4, CD8, S100 | Mouse | Positive/ Negative | Mean no cells/5 x400 fields  CD4 >20 = +  CD8 >100 = + | - | - | (0) | (0) | OS | Tumour ≥3.5cm, N stage, margin involvement, LVI, CD4/CD8 (+/+) |

- Data not recorded, ANT adjacent normal tissue, C cytoplasm, CEA carcinoembryonic antigen, DFS disease free survival, DMFS distant metastasis free survival, DSS disease specific survival, I tumour islet, IE intraepithelial, IF invasive front, GC germinal centre, LPFS local progression free survival, LVI lymphovascular invasion, M membranous, mDC mature dendritic cells, MHC1 major histocompatibility class 1, MVD micro vessel density, NLR neutrophil lymphocyte ratio, OS overall survival, P periphery, plx nerve plexus, PNI perineural invasion, PV perivascular, Q quartile, ref reference value, S stroma, TC tumour core, TLT tertiary lymphoid tissue, Treg regulatory T cells.

| **Table S3.** REMARK score | | | | | | | | | | | |
| --- | --- | --- | --- | --- | --- | --- | --- | --- | --- | --- | --- |
| **Author** | **Intro** | **Patients** | **Specimen** | **Assay** | **Design** | **Stats** | **Data** | **Analysis** | **Discussion** | **Total** |  |
|  |  | **(2)** |  |  | **(4)** | **(2)** | **(2)** | **(5)** | **(2)** | **(20)** |  |
| **Mahajan (2018)** | 1 | 2 | 1 | 1 | 4 | 2 | 2 | 5 | 2 | 20 |  |
| **Tahkola (2018)** | 1 | 2 | 1 | 1 | 3 | 2 | 1 | 4 | 2 | 18 |  |
| **Wartenberg (2018)** | 1 | 1 | 1 | 1 | 2 | 2 | 1 | 1 | 2 | 12 |  |
| **Balachandran (2017)** | 1 | 1 | 1 | 1 | 4 | 2 | 2 | 4 | 2 | 18 |  |
| **Imai (2017)** | 1 | 1 | 1 | 1 | 2 | 2 | 2 | 1 | 2 | 13 |  |
| **Lohneis (2017)** | 1 | 2 | 1 | 1 | 4 | 2 | 1 | 1 | 2 | 15 |  |
| **Nejati (2017)** | 1 | 1 | 1 | 1 | 3 | 2 | 2 | 4 | 2 | 17 |  |
| **Tessier-Cloutier (2017)** | 1 | 2 | 1 | 1 | 3 | 2 | 2 | 4 | 2 | 18 |  |
| **Zhang (2017)** | 1 | 2 | 1 | 1 | 3 | 2 | 1 | 3 | 2 | 16 |  |
| **Castino (2016)** | 1 | 2 | 1 | 1 | 3 | 2 | 2 | 4 | 2 | 18 |  |
| **Diana CD8 (2016)** | 1 | 2 | 1 | 1 | 3 | 2 | 2 | 4 | 2 | 18 |  |
| **Diana CA IX (2016)** | 1 | 2 | 1 | 1 | 3 | 2 | 1 | 4 | 2 | 17 |  |
| **Hu (2016)** | 1 | 2 | 1 | 1 | 2 | 2 | 1 | 4 | 2 | 16 |  |
| **Hutcheson (2016)** | 1 | 2 | 1 | 1 | 2 | 2 | 1 | 3 | 2 | 15 |  |
| **Hwang (2016)** | 1 | 2 | 1 | 1 | 3 | 2 | 1 | 2 | 2 | 15 |  |
| **Liu (2016)** | 1 | 2 | 1 | 1 | 2 | 2 | 2 | 4 | 2 | 17 |  |
| **Takakura (2016)** | 1 | 2 | 1 | 1 | 1 | 1 | 1 | 3 | 2 | 13 |  |
| **Wang (2016)** | 1 | 2 | 1 | 1 | 2 | 2 | 2 | 4 | 2 | 17 |  |
| **Tang (2015)** | 1 | 2 | 1 | 1 | 2 | 2 | 1 | 4 | 2 | 16 |  |
| **Chen (2014)** | 1 | 2 | 1 | 1 | 1 | 1 | 1 | 2 | 2 | 12 |  |
| **Sugimoto (2014)** | 1 | 2 | 1 | 1 | 3 | 2 | 2 | 4 | 2 | 18 |  |
| **Tewari (2013)** | 1 | 2 | 1 | 1 | 2 | 1 | 2 | 2 | 2 | 14 |  |
| **Kurahara (2012)** | 1 | 2 | 1 | 1 | 2 | 1 | 1 | 1 | 2 | 12 |  |
| **Yoshikawa (2012)** | 1 | 1 | 1 | 1 | 1 | 2 | 1 | 3 | 2 | 13 |  |
| **Hiraoka (2006)** | 1 | 2 | 1 | 1 | 3 | 2 | 2 | 4 | 2 | 18 |  |
| **Fukunaga (2004)** | 1 | 2 | 1 | 1 | 3 | 2 | 1 | 4 | 2 | 17 |  |

| **Table S4.** Newcastle-Ottawa score | | | | | | | | | |
| --- | --- | --- | --- | --- | --- | --- | --- | --- | --- |
| **Author** | **Selection** |  |  |  | **Comparability** | **Outcome** |  |  | **Total (9)** |
|  | Representativeness of exposed cohort | Selection of non-exposed cohort | Ascertainment of exposure | Outcome not present at start of study | of cohorts on basis of design | Assessment of outcome | Was follow-up long enough | Was follow-up complete |  |
| **Mahajan (2018)** | ***** | ***** | ***** | ***** | ****** | ***** | ***** | ***** | 9 |
| **Tahkola (2018)** | ***** | ***** | ***** | ***** | ****** | ***** | ***** | ***** | 9 |
| **Wartenberg (2018)** | ***** | ***** | ***** | ***** | **- -** | ***** | ***** | ***** | 7 |
| **Balachandran (2017)** | ***** | ***** | ***** | ***** | ****** | ***** | ***** | ***** | 9 |
| **Imai (2017)** | ***** | ***** | ***** | ***** | **- -** | ***** | ***** | ***** | 7 |
| **Lohneis (2017)** | ***** | ***** | ***** | ***** | ****** | ***** | ***** | ***** | 9 |
| **Nejati (2017)** | ***** | ***** | ***** | ***** | ****** | ***** | ***** | ***** | 9 |
| **Tessier-Cloutier (2017)** | ***** | ***** | ***** | ***** | ****** | ***** | ***** | ***** | 9 |
| **Zhang (2017)** | ***** | ***** | ***** | ***** | ****** | ***** | ***** | ***** | 9 |
| **Castino (2016)** | ***** | ***** | ***** | ***** | ****** | ***** | ***** | ***** | 9 |
| **Diana CD8 (2016)** | * | * | * | * | ** | * | * | * | 9 |
| **Diana CA IX (2016)** | ***** | ***** | ***** | ***** | ****** | ***** | ***** | ***** | 9 |
| **Hu (2016)** | ***** | ***** | ***** | ***** | ****** | ***** | ***** | ***** | 9 |
| **Hutcheson (2016)** | * | * | * | * | ** | * | * | * | 9 |
| **Hwang (2016)** | ***** | ***** | ***** | ***** | ****** | ***** | ***** | ***** | 9 |
| **Liu (2016)** | ***** | ***** | ***** | ***** | ****** | ***** | ***** | ***** | 9 |
| **Takakura (2016)** | ***** | ***** | ***** | ***** | ****** | ***** | ***** | ***** | 9 |
| **Wang (2016)** | ***** | ***** | ***** | ***** | ****** | ***** | ***** | ***** | 9 |
| **Tang (2015)** | ***** | ***** | ***** | ***** | ****** | ***** | ***** | ***** | 9 |
| **Chen (2014)** | ***** | ***** | ***** | ***** | ****** | ***** | ***** | ***** | 9 |
| **Sugimoto (2014)** | ***** | ***** | ***** | ***** | ****** | ***** | ***** | ***** | 9 |
| **Tewari (2013)** | ***** | ***** | ***** | ***** | **- -** | ***** | ***** | ***** | 7 |
| **Kurahara (2012)** | ***** | ***** | ***** | ***** | ****** | ***** | ***** | ***** | 9 |
| **Yoshikawa (2012)** | ***** | ***** | ***** | ***** | ****** | ***** | ***** | ***** | 9 |
| **Hiraoka (2006)** | ***** | ***** | ***** | ***** | ****** | ***** | ***** | ***** | 9 |
| **Fukunaga (2004)** | ***** | ***** | ***** | ***** | ****** | ***** | ***** | ***** | 9 |
